# Supplementary material for: A two-phase transfer learning framework for gastrointestinal diseases classification
Source: PeerJ Comput Sci. 2024 Dec 19;10:e2587. doi: 10.7717/peerj-cs.2587 (PMC11784777; doi:10.7717/peerj-cs.2587)
Supplement: Supplemental Information 1 [file peerj-cs-10-2587-s001.docx]

- **Code for reproducibility: Gastrointestinal-Diseases-Classification-main**

A GitHub repository containing all the code has been made available at <https://github.com/byahmedali/Gastrointestinal-Diseases-Classification.git>

- The code was developed using the free hardware resources provided by Google Colab. You can access Google Colab here: <https://colab.research.google.com/?hl=en-GB>
- A README file, detailing the code and usage instructions, is also available in the same GitHub repository.
